# Exercise ameliorates hepatic lipid accumulation via upregulating serum PRL and activating hepatic PRLR-mediated JAK2/STAT5 signaling pathway in NAFLD mice

**DOI:** 10.3389/fphar.2025.1647231

**Published:** 2025-08-14

**Authors:** Jia Fan, Kaidi Nie, Xiaobing Liu, Jiao Liu, Binghua Shao

**Affiliations:** ^1^ Institute of Sports Medicine and Health, Chengdu Sport University, Chengdu, China; ^2^ School of Health Preservation and Rehabilitation, Chengdu University of Traditional Chinese Medicine, Chengdu, Sichuan, China; ^3^ School of Physical Education and Health, Chengdu University of Traditional Chinese Medicine, Chengdu, China

**Keywords:** non-alcoholic fatty liver disease (NAFLD), exercise, hepatic lipid accumulation, serum PRL, JAK/Stat5

## Abstract

**Objective:**

Non alcoholic fatty liver disease (NAFLD) is one of the most common chronic liver diseases, closely related to overnutrition, obesity, and metabolic syndrome. This study used exercise intervention on NAFLD mice to investigate the effect of aerobic exercise on NAFLD.

**Method:**

After one week of acclimatization, the mice were randomly divided into two groups: a control group (C group, n = 14) fed a standard diet and a model group (M group, n = 24) fed a high-fat diet. At the end of the 10th week, four mice from each group were randomly selected for liver pathological sectioning and Oil Red O staining to assess hepatic lipid droplet formation and confirm the successful establishment of the NAFLD model. Subsequently, the remaining mice in M group were further randomized into two subgroups: a model control group (CM group, n = 10) and a model exercise group (EM group, n = 10). After modeling, the blood glucose tolerance of mice, HE staining and red oil staining of liver tissue, HDL-C, LDL, TC, TG, AST, ALT, PRL content in serum, and JAK, PRLR, STAT5 content in liver were detected.

**Result:**

The glucose tolerance test found that at 10 and 30 minutes, compared with the C group, the blood glucose level in the CM group increased (P < 0.05), while the blood glucose level in the EM group decreased compared to the CM group (P < 0.05); After 60 minutes, there was no statistically significant difference in blood glucose levels among the groups of rats compared to C group, the final body weight of CM group and EM group was significantly higher (P < 0.01). The final body weight of EM group was significantly lower than that of CM group (P < 0.01). In terms of liver index, both C group and EM group showed significantly lower values than CM group (P < 0.01). Compared to C group, both CM group and EM group exhibited significantly higher levels of TC and LDL-C (P < 0.01) whereas HDL-C levels were significantly lower in CM group (P < 0.01). When compared to CM group, EM group showed significantly reduced LDL-C levels (P < 0.01), while HDL-C levels were significantly higher (P < 0.01). Compared with group C, there were significant differences in serum AST, ALT, and PRL levels in the CM group (P < 0.01). There were significant differences in serum AST, ALT, and PRL levels between the EM group and the CM group (P < 0.05).The gene testing results of JAK and STAT5a showed no statistical difference in expression among the three groups. The PRLR gene results showed that compared with the C group, the PRLR in the CM group was significantly reduced (p < 0.01); Compared with the CM group, the PRLR in the CE group was significantly increased (p < 0.01). Compared with group C, the expression levels of PRLR and p-STAT5 were significantly reduced in both CM and EM groups (p < 0.01). The p-JAK2 levels in the CM group were significantly reduced compared to the C group (p < 0.01). The expression levels of PRLR, p-JAK2, and p-STAT5 in the EM group were significantly higher than those in the CM group (p < 0.01).

**Conclusion:**

Exercise may moderately elevate serum prolactin (PRL) levels, thereby reducing intrahepatic lipid accumulation and ameliorating non-alcoholic fatty liver disease (NAFLD). The underlying mechanism may involve upregulation of the hepatic classical PRLR-mediated JAK2/STAT5 signaling pathway.

## 1 Introduction

Non-alcoholic fatty liver disease (NAFLD) is a globally prevalent liver disorder, affecting approximately 25% of the adult population. Importantly, NAFLD is also frequently observed in lean individuals, with an estimated prevalence of 16% in this demographic (Loomba et al., 2021). NAFLD is a multifactorial condition characterized by the abnormal accumulation of lipids in liver tissue in absence of secondary causes, including significant alcohol consumption, chronic use of medications and hereditary disorders (Cataldo et al., 2021). NAFLD is typically associated with overnutrition, obesity, and manifestations related to metabolic syndrome (Loomba et al., 2021). A large body of clinical evidence indicates that NAFLD is associated not only with increased liver-related morbidity and mortality, but also with an increased risk of developing other important extra-hepatic diseases, such as cardiovascular disease, (that is the predominant cause of death in patients with NAFLD (Kasper et al., 2021)), extra-hepatic cancers (mainly colorectal cancers), T2DM and chronic kidney disease (Mantovani et al., 2020). Research indicates that an unreasonable dietary structure, particularly a high-fat diet, coupled with a sedentary lifestyle, are significant contributing factors to the development of NAFLD (Heredia et al., 2022). While weight reduction through dietary interventions or bariatric surgery has proven effective in managing NAFLD, there are currently no drugs specifically approved for its treatment (Sumida and Yoneda, 2018). Therefore, lifestyle modifications serve as a cornerstone in the effective management of NAFLD patients (Hao et al., 2025), positioning exercise as a pivotal intervention in both the prevention and treatment of NAFLD. This has established exercise as a prominent research focus within the fields of clinical medicine and sports medicine.

Prolactin (PRL) is a polypeptide hormone predominantly synthesized and secreted by specialized cells in the anterior pituitary gland but also by other peripheral tissues (Chasseloup et al., 2024). The prolactin receptor (PRLR), a transmembrane receptor, is a member of the type I cytokine receptor superfamily (Gorvin, 2015). PRL binds directly to a unique transmembrane receptor (PRLR), and the Janus kinase 2 (JAK2)/signal transducer and activator of transcription 5 (Stat5) pathway is considered the major downstream pathway for PRLR signaling (Chasseloup et al., 2024). Previous studies have demonstrated that the JAK2/STAT5 signaling pathway plays a critical role in lipid metabolism (Barclay et al., 2011; Li et al., 2019; Zhang et al., 2020). However, large cohort clinical studies have recently shown that low circulating PRL levels are associated with metabolic disease, whereas high PRL levels acts on the pancreas, liver, adipose tissue, and hypothalamus to maintain and promote metabolic homeostasis (Macotela et al., 2022; Macotela et al., 2020).

Effective prevention of NAFLD through exercise is closely associated with the reduction of visceral lipid accumulation and regulation of lipid metabolism. It has been hypothesized that aerobic exercise may alleviate hepatic lipid accumulation by elevating circulating prolactin (PRL) levels, although the precise underlying mechanisms remain unclear. In this study, we systematically evaluated the effects of aerobic exercise on body weight, liver wet weight, liver index, serum lipid profiles, histopathological changes in liver tissue, and circulating PRL levels in a murine NAFLD model. Furthermore, we demonstrated that aerobic exercise ameliorates hepatic lipid accumulation through upregulation of circulating PRL levels and subsequent activation of the primary downstream signaling pathways of the PRLR in the liver. These findings provide novel insights into the mechanisms underlying the preventive and therapeutic effects of exercise on NAFLD, offering potential strategies for clinical intervention.

## 2 Materials and methods

### 2.1 Animal experiments and grouping

Thirty-eight 6-week-old male C57BL/6 mice, weighing 19.9 ± 0.80 g, were purchased from Shanghai Southern Model Biotechnology Co., Ltd. [Production License No.: SCXK (Hu) 2017-0010]. The mice were housed and subjected to exercise training in the SPF-level animal facility of Chengdu Sport University [Animal Use License No.: SYXK (Chuan) 2018-211]. Animal experiments were conducted in compliance with the regulations of the Laboratory Animal Ethics Committee of Chengdu Sport University (approved No.:202159, approved date: 29 October 2021). The mice were housed in separate cages with *ad libitum* access to food and water, under controlled environmental conditions (room temperature: 20°C ± 2°C; relative humidity: 45%–55%) with a simulated light-dark cycle. After 1 week of acclimatization, the mice were randomly divided into two groups: a control group (C group, n = 14) fed a standard diet and a model group (M group, n = 24) fed a high-fat diet. At the end of the 10th week, four mice from each group were randomly selected for liver pathological sectioning and Oil Red O staining to assess hepatic lipid droplet formation and confirm the successful establishment of the NAFLD model. Subsequently, the remaining mice in M group were further randomized into two subgroups: a model control group (CM group, n = 10) and a model exercise group (EM group, n = 10).

### 2.2 Diets and formulations

Both the standard diet and the high-fat diet were formulated by Jiangsu Synergetic Medical Bioengineering Co., Ltd. [License No.: Su Si Zheng (2019) 01008]. The high-fat diet was composed of the following ingredients by weight: 38% basal diet, 28% lard, 5.6% sucrose, 10.8% whole milk powder, 11.5% casein, 1.9% microcrystalline cellulose, 2% experimental animal premix, 1.8% calcium hydrogen phosphate, and 0.4% limestone. The macronutrient energy distribution of the high-fat diet was as follows: 18.14% protein, 60.55% fat, and 21.22% carbohydrates.

### 2.3 Exercise protocol

The exercise protocol was adapted from the methodology described by Zhu et al. (2021), with necessary modifications to accommodate the specific experimental conditions of our animal study. The exercise training regimen consisted of treadmill running at a 0° incline. The experimental group underwent a moderate-intensity treadmill training program, commencing at an initial speed of 10 m/min for 20 min daily. The training intensity was progressively increased by 0.5 m/min each day until reaching 14 m/min, while the duration was extended by 10 min daily until achieving 60 min per session. This training protocol was maintained for 5 consecutive days per week over an 8-week period.

### 2.4 Tissue collection

Twenty-four hours after the completion of the experiment, the mice were weighed and anesthetized via intraperitoneal injection of 4 mL/kg of 2% sodium pentobarbital solution. Blood samples were collected through the ocular vein, and the abdominal cavity was disinfected and opened to rapidly excise the liver. The liver was repeatedly rinsed in ice-cold physiological saline (4°C). A 1 cm × 1 cm×1 cm section from the middle portion of the left lobe was collected, divided into cryotubes, and immediately snap-frozen in liquid nitrogen for subsequent Oil Red O staining and Western blot analysis. A portion of the right lobe was fixed in 4% paraformaldehyde for HEstaining.

### 2.5 Biochemical analyses

#### 2.5.1 Serum lipid profile

The levels of total cholesterol (TC), triglycerides (TG), high-density lipoprotein cholesterol (HDL-C), and low-density lipoprotein cholesterol (LDL-C) in serum were measured using an automated biochemical analyzer.

#### 2.5.2 Sugar tolerance test

Fasting for 16 h on the first day before the test, and intraperitoneal injection of 50% high glucose solution (2.0 g/kg) on the second day. Blood samples were collected from the tail vein at 0, 10, 30, 60, and 120 min after injection, and blood glucose levels were measured using a blood glucose meter.

#### 2.5.3 HE staining

Paraffin-embedded sections (5 μm) were prepared and subjected to conventional HE staining. During the staining process, lipid deposits in hepatocytes were removed through deparaffinization, resulting in vacuolated appearances under light microscopy, which were used to assess the pathomorphological features of NAFLD. A double-blind evaluation was conducted by two independent pathologists. Based on the method described in the literature (Chinese Society of Hepatology, Chinese Medical Association, 2024), the extent of hepatic steatosis was quantified using a scoring system. The scoring criteria were as follows: 0 points for scattered and sparse fatty droplets in hepatocytes; 1 point for fatty droplets occupying ≤25% of the hepatocyte area; 2 points for fatty droplets occupying ≤50% of the hepatocyte area; 3 points for fatty droplets occupying ≤75% of the hepatocyte area; and 4 points for fatty droplets replacing almost the entire liver tissue, with >75% of the hepatocyte area affected.

#### 2.5.4 Oil Red O staining

Frozen sections (5 μm) were prepared and fixed in formaldehyde-calcium solution for 10 min, followed by thorough rinsing with distilled water. The sections were then immersed in 60% isopropanol and stained with Oil Red O solution for 10 min under dark conditions. Excess stain was removed by differentiation in 60% isopropanol until the background became colorless. After another thorough rinse with distilled water, the sections were counterstained with Mayer’s hematoxylin, washed under running tap water for 1–3 min, and rinsed again with distilled water. Finally, the sections were mounted with glycerin gelatin. For each slide, five random fields were examined under a light microscope at ×200 magnification. The Image-Pro Plus 6.0 image analysis system was used to perform semi-quantitative analysis, calculating the average optical density (expressed as AIOD/μm^2^) of red lipid droplets in each field of view.

#### 2.5.5 Enzyme-linked immunosorbent assay (ELISA)

The double-antibody sandwich enzyme-linked immunosorbent assay (ELISA) was performed strictly according to the manufacturer’s instructions. Use mouse AST, ALT, and PRL ELISA kits to quantitatively detect their expression levels.

#### 2.5.6 Western blot

Approximately 20 mg of liver tissue was homogenized in RIPA lysis buffer supplemented with PMSF (1 mg tissue/20 μL lysis buffer) to extract total protein. The protein concentration was determined using the BCA assay. A mixture of loading buffer and protein (1:1 ratio) was denatured by heating in a 100°C water bath for 10 min. After cooling, the samples were aliquoted and stored at −20°C. Protein samples were separated by SDS-PAGE electrophoresis and subsequently transferred onto a polyvinylidene difluoride (PVDF) membrane alongside a molecular weight marker. The PVDF membrane was blocked with 5% bovine serum albumin (BSA) at room temperature for 1 h and then incubated overnight at 4°C with gentle shaking in the presence of the following primary rabbit monoclonal antibodies: total PRLR (1:500 dilution, ER 1915-43, Huabio), JAK2 (1:1000 dilution, ET1607-35, Huabio), phosphorylated JAK2 (p-JAK2; 1:1000 dilution, ET1607-34, Huabio), STAT5a (1:1000 dilution, ET1610-58, Huabio), phosphorylated STAT5 (p-STAT5; 1:1000 dilution, ET1610-48, Huabio), and β-actin (1:100,000 dilution, AC026, Abclonal). The following day, the membrane was washed with TBST buffer (8 × 5 min) and incubated with a horseradish peroxidase (HRP)-conjugated goat anti-rabbit secondary antibody (1:5000 dilution, ab6721, Abcam) at room temperature for 2 h, followed by another round of TBST washes (8 × 5 min).

Protein bands were visualized using enhanced chemiluminescence (ECL) substrate and imaged with a chemiluminescence gel imaging system. Densitometric analysis of the blots was performed using Quantity One software, and the results were normalized to β-actin expression. The relative protein levels were expressed as a percentage of the control group.

#### 2.5.7 RT-PCR

Total RNA was extracted from liver cells, reverse transcribed into cDNA according to the kit operation procedure, added to the amplification reaction system, and subjected to qRT PCR detection using GAPDH as an internal reference. The results were compared for relative expression using the 2^-Δ^ ^ΔCT^ method.

### 2.6 Statistical analysis

All data are presented as mean ± standard deviation (SD), unless otherwise specified. Statistical analyses were performed using SPSS 19.0 software. Differences between groups were assessed by one-way analysis of variance (ANOVA), followed by the least significant difference (LSD) test for comparisons under the assumption of homogeneity of variance. In cases of unequal variance, Tamhane’s T2 test was applied. A P-value of <0.01 was considered statistically significant.

## 3 Results

### 3.1 General health status

**FIGURE 1:**
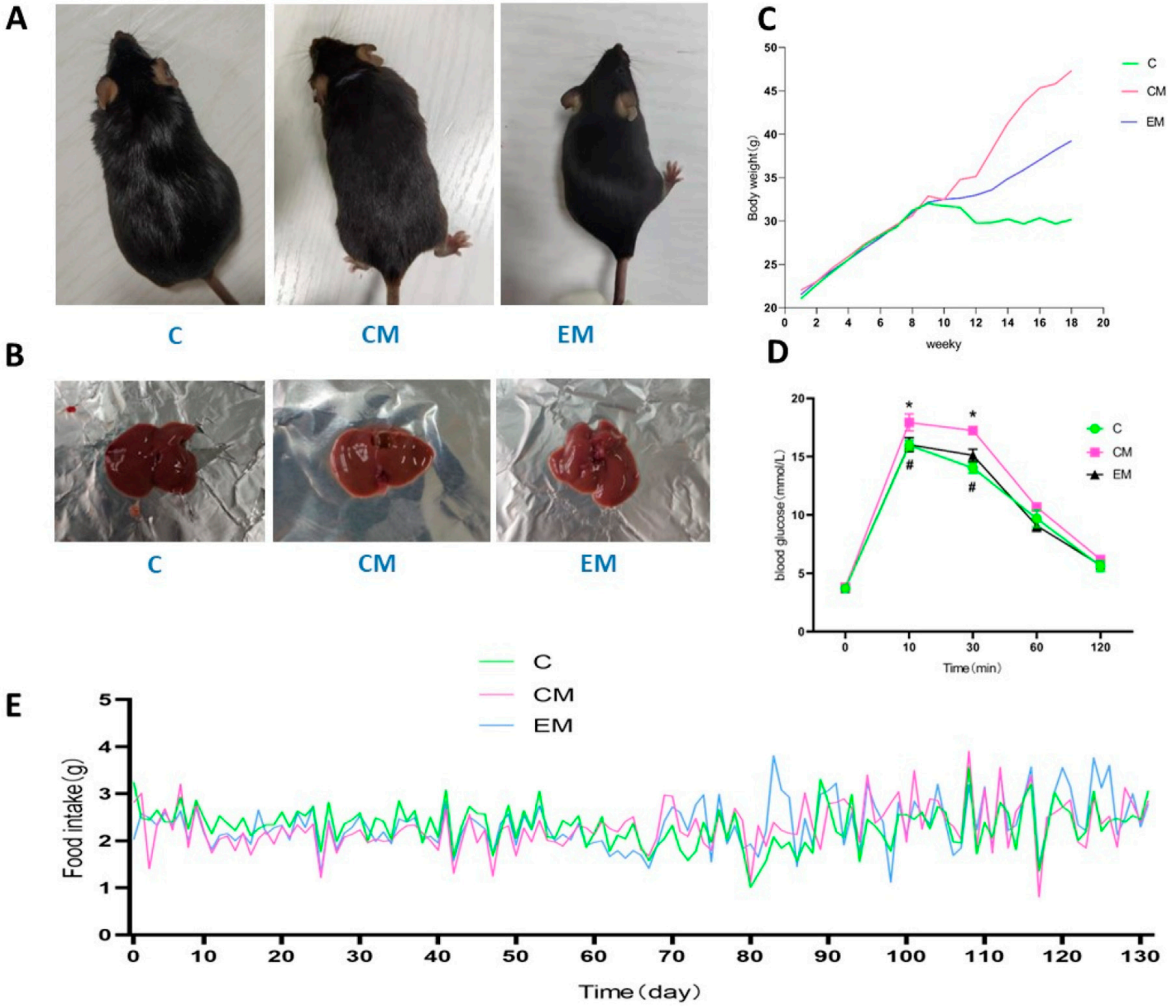
Mice appearance **(A)**, Physical image of rat liver **(B)**, Trend of body weight changes **(C)**, Results of glucose tolerance test **(D)**, Daily feed intake of mice **(E)**.

Mice in C group exhibited shiny fur, high activity levels, and overall agility. In contrast, mice in CM group displayed patchy, yellowish, and dull fur, along with signs of lethargy, reduced mobility, and significant weight gain. Notably, the general health status of EM group showed marked improvement compared to that of CM group (Figure 1A).

### 3.2 Body weight changes

Body weight in all groups increased steadily during the first 9 weeks. From the 9th week onward, the rate of weight gain in CM group and EM group was significantly higher than that in C group. Among these groups, the fastest weight gain was observed in CM group, followed by EM group, while C group exhibited the slowest rate of weight increase (Figure 1C). During the 131 days experiment, the daily food intake of each mouse in each group was recorded (Figure 1E).

#### 3.2.1 3sugar tolerance

The glucose tolerance test found that at 10 and 30 min, compared with the C group, the blood glucose level in the CM group increased (P < 0.05), while the blood glucose level in the EM group decreased compared to the CM group (P < 0.05); After 60 min, there was no statistically significant difference in blood glucose levels among the groups of rats (Figure 1D).

### 3.3 Changes in Liver Index

**FIGURE 2:**
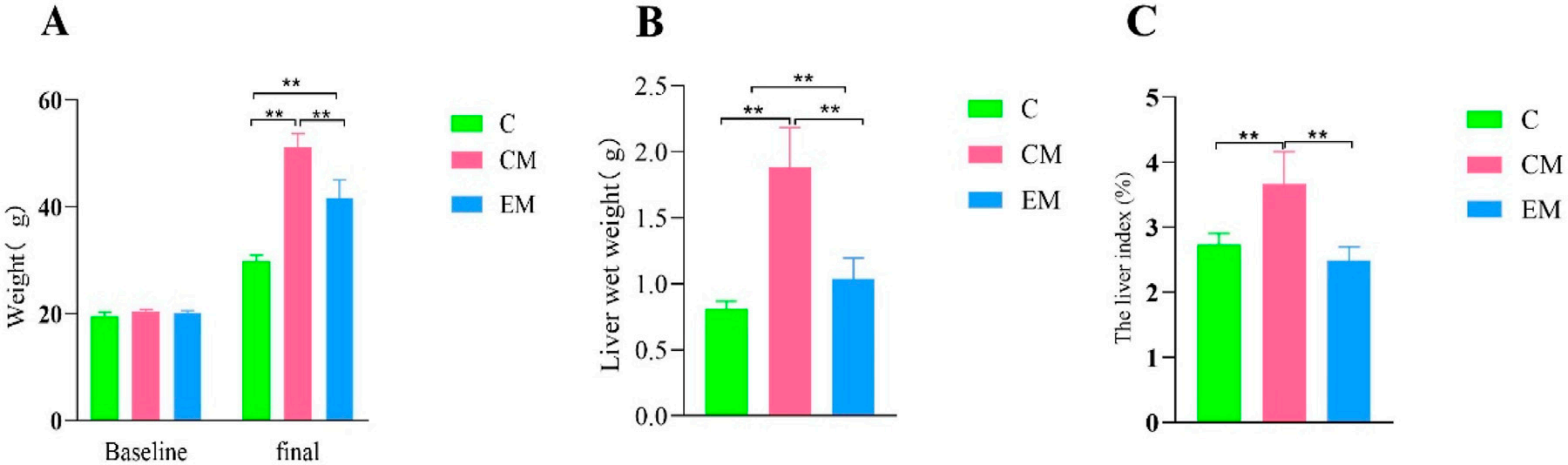
Changes in Liver Index. **(A)** Baseline and final body weight measurements. Baseline body weight was recorded after 1 week of adaptive feeding, while final body weight was measured at the time of sacrifice. **(B)** Liver wet weight measured at the time of sacrifice. **(C)** Liver index (%) calculated as the ratio of liver wet weight to final body weight.

The liver solid images of each group of rats (Figure 1B). No significant differences were observed in baseline body weight among the groups; however, significant differences were noted in final body weight (Figure 2A). Specifically, compared to C group, the final body weight of CM group and EM group was significantly higher (*P* < 0.01). Notably, the final body weight of EM group was significantly lower than that of CM group (*P* < 0.01). Similar trends were observed in liver wet weight compared to final body weight (Figure 2B). In terms of liver index, both C group and EM group showed significantly lower values than CM group (*P* < 0.01) (Figure 2C).

### 3.4 Serum lipid profiles

**FIGURE 3:**
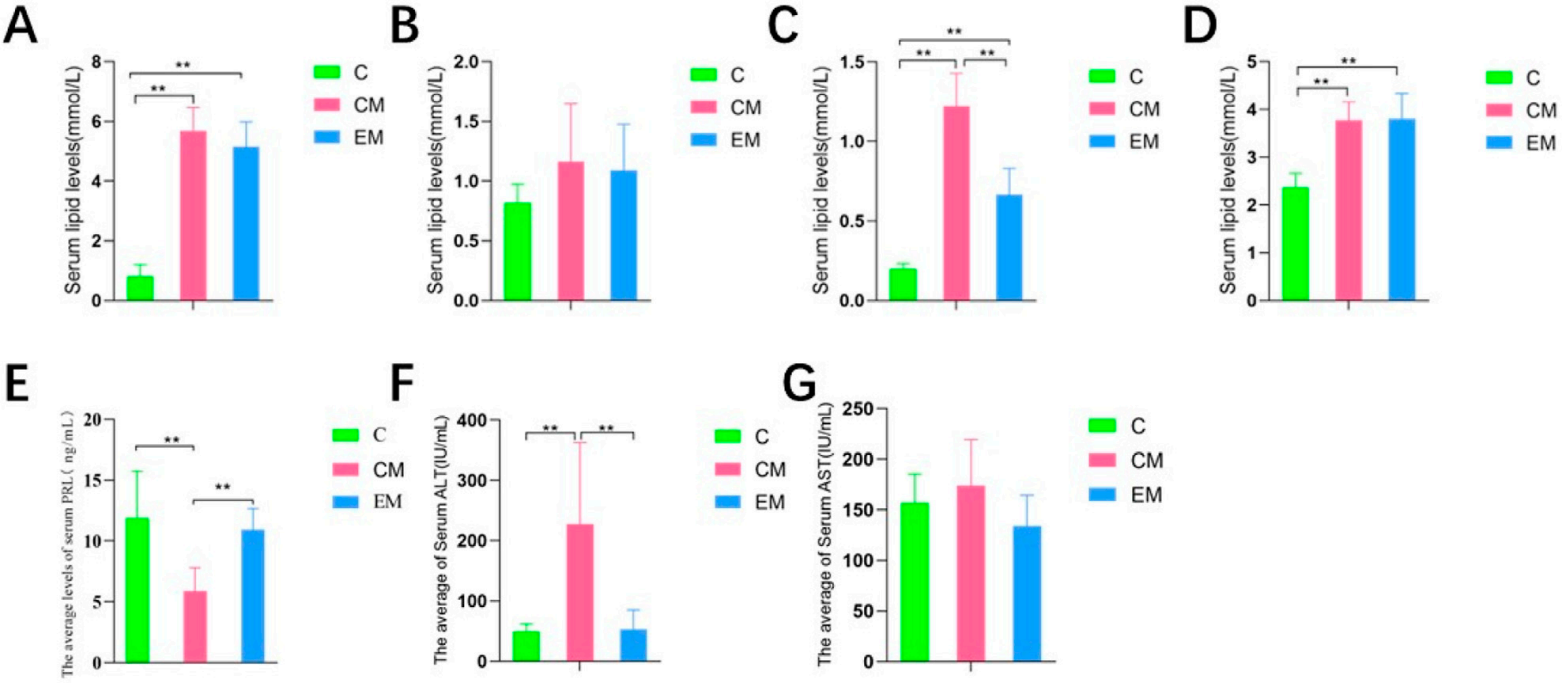
Serum levels of total TC **(A)**, TG **(B)**, LDL-C **(C)** and HDL-C **(D)** in mice. PRL levels in mice. **(E)** ALT **(F)**, AST **(G)** levels in mice.

With the exception of HDL-C, the trends for other serum lipid indicators were consistent across groups: CM > EM > C (Figure 3D). Compared to C group, both CM group and EM group exhibited significantly higher levels of TC and LDL-C (*P* < 0.01) (Figure 3A,C), whereas HDL-C levels were significantly lower in CM group (*P* < 0.01). Similarly, when compared to CM group, EM group showed significantly reduced LDL-C levels (*P* < 0.01), while HDL-C levels were significantly higher (*P* < 0.01).

### 3.5 Serum AST, ALT, and PRL *levels*

The results of serum AST and ALT detection in each group of mice: Compared with group C (Figure 3G), the serum AST and ALT levels in the CM group were significantly increased (P < 0.01) (Figure 3F). In contrast, the serum AST and ALT levels in the EM group were significantly lower than those in the CM group (P < 0.05). The results of serum PRL detection in each group of mice showed that compared with group C (Figure 3E), the serum PRL level in the CM group was significantly reduced (P < 0.01). In contrast, the serum PRL levels in the EM group were significantly higher than those in the CM group (P < 0.05).

### 3.6 Expression of proteins in PRLR-mediated JAK2/STAT5 signaling pathway in liver tissue

**FIGURE 4:**
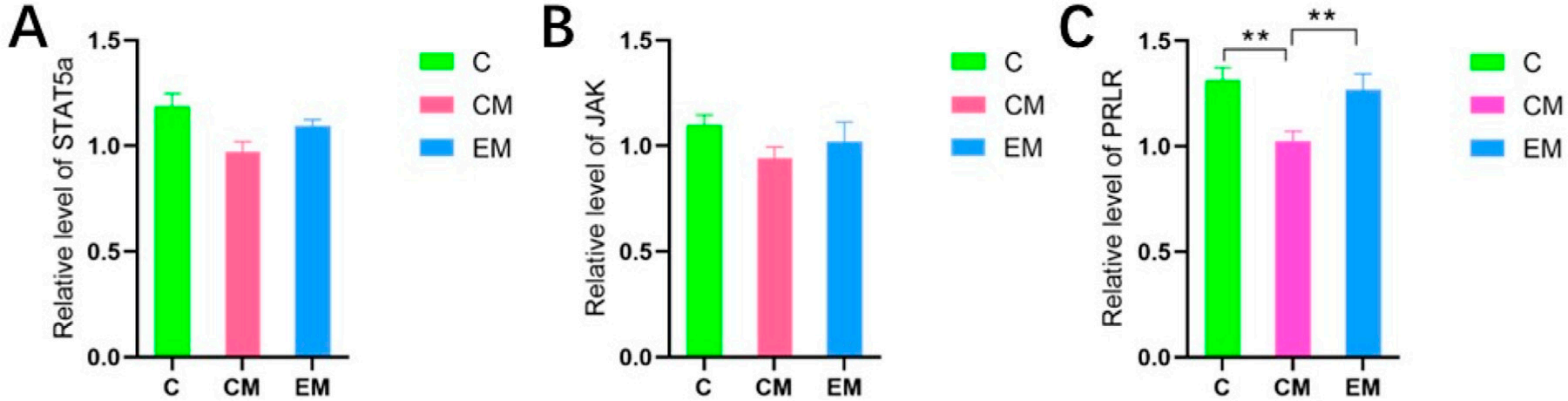
**(A)** Results of RT-PCR detection of STAT5a, **(B)** Results of RT-PCR detection of JAK, **(C)** Results of RT-PCR detection of PRLR.

The gene testing results of JAK and STAT5a showed (Figures 4A,B) that there was no statistical difference in expression among the three groups. The gene results of PRLR showed (Figure 4C) that compared with group C, PRLR was significantly reduced in the CM group (p < 0.01); Compared with the CM group, PRLR was significantly increased in the CE group (p < 0.01).

### 3.7 Hepatic pathological changes

**FIGURE 5:**
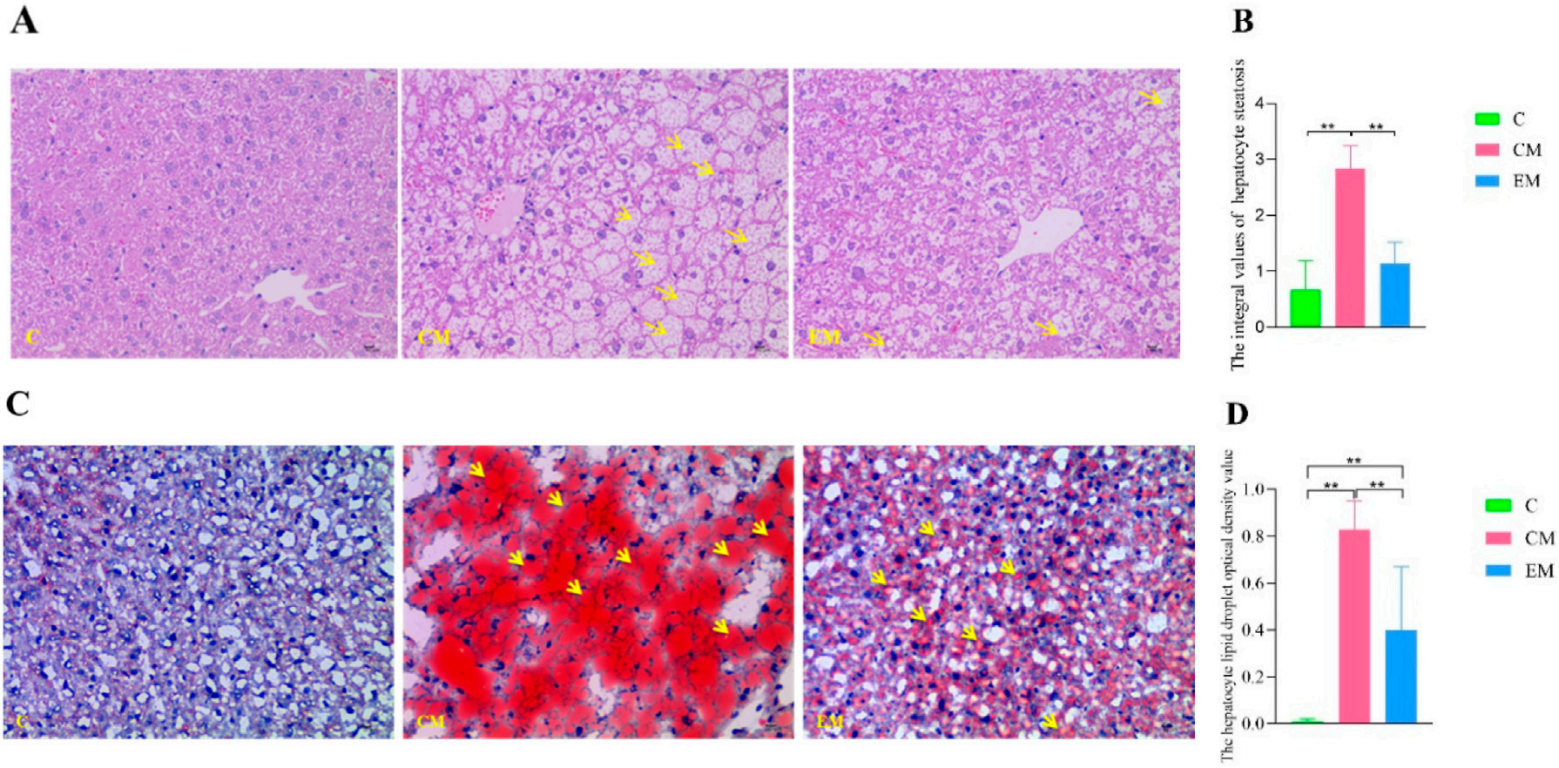
Hepatic Pathological Changes. **(A)** Representative images of hepatocyte steatosis assessed by HE staining. Yellow arrows indicate fatty vacuoles (×400 magnification; scale bar = 10 µm). **(B)** Quantitative analysis of hepatocyte steatosis scores in liver tissues, evaluated by two independent pathologists using light microscopy according to the method described in the literature (Chinese Society of Hepatology, Chinese Medical Association, 2024). **(C)** Representative images of lipid droplets in liver tissues visualized by Oil Red O staining. Yellow arrows indicate red lipid droplets (×400 magnification; scale bar = 10 µm). **(D)** Quantitative analysis of lipid droplet optical density in liver tissues, measured using Image-Pro Plus 6.0 software.

HE Staining Results (Figure 5A): In C group, no hepatocyte steatosis was observed, with hepatocytes arranged in neat rows and round nuclei centrally located. In contrast, CM group exhibited severe hepatocyte steatosis, characterized by disordered cell arrangement, nuclei displaced to the cell periphery due to the compression from lipid droplets, and numerous fatty vacuoles scattered throughout the cytoplasm. Compared to CM group, EM group showed a significant reduction in steatosis, with hepatocytes more neatly arranged and smaller lipid vacuoles.

Quantification of Hepatocyte Steatosis (Figure 5B): The steatosis scores in CM group were significantly higher than those in C group (*P* < 0.01), whereas EM group demonstrated significantly lower scores compared to CM group (*P* < 0.01).

Oil Red O Staining Results (Figure 5C): In C group, only a few hepatocytes were stained red, indicating minimal lipid deposition. In CM group, hepatocytes exhibited extensive red staining of varying sizes, reflecting widespread intracellular lipid droplet accumulation. In comparison, EM group showed significantly fewer red-stained hepatocytes, suggesting reduced lipid deposition.

Quantification of Lipid Droplet Optical Density (Figure 5D): Consistent with the HE staining results, the optical density values of lipid droplets in CM group were significantly higher than those in C group (*P* < 0.01), while EM group displayed significantly lower values compared to CM group (*P* < 0.01).

**FIGURE 6:**
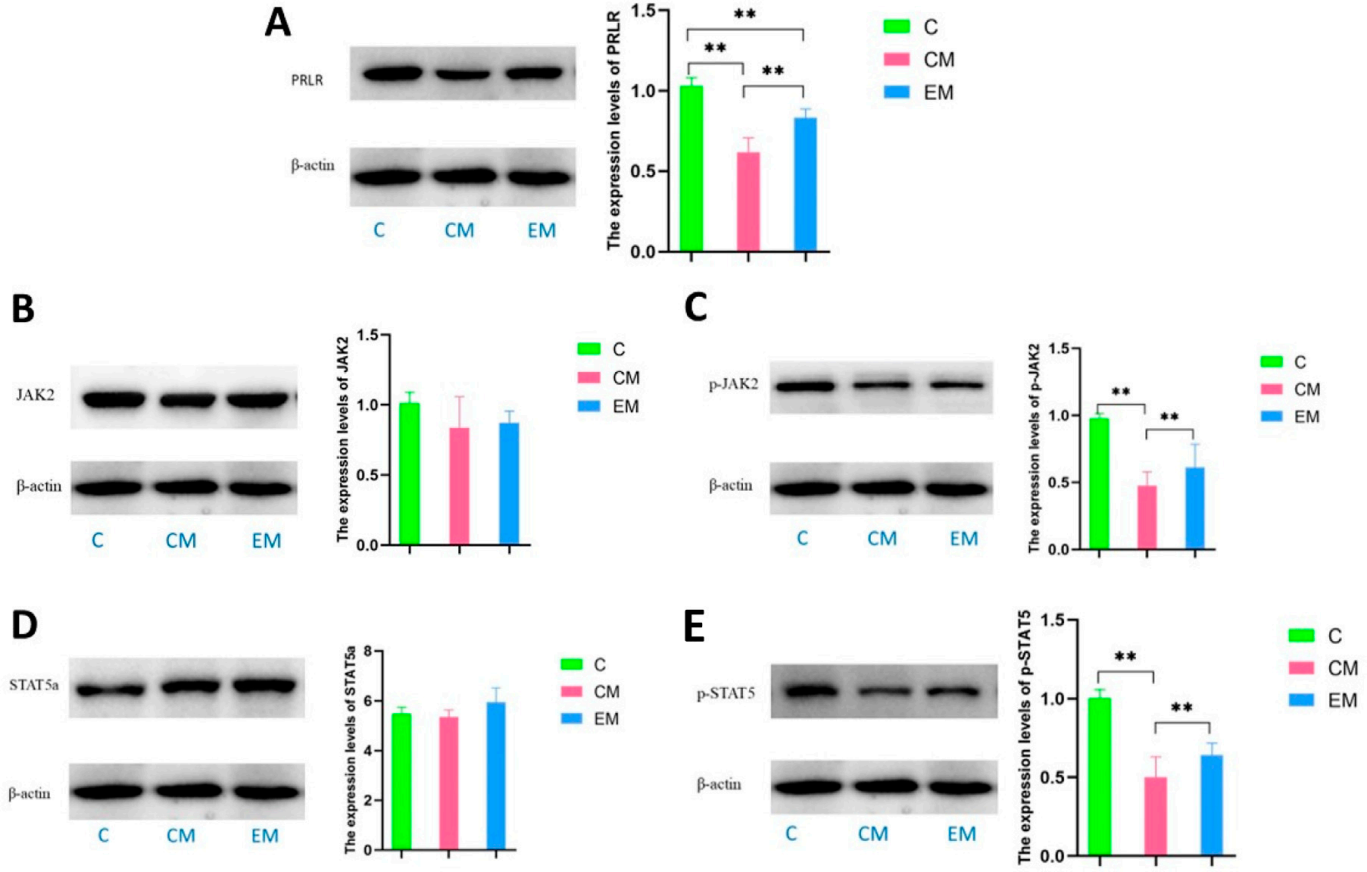
Expression of PRL/PRLR signaling pathway related proteins in liver tissue, and expression levels of PRLR **(A)**, JAK2 **(B)**, p-JAK2 **(C)**, STAT5a **(D)**, and p-STAT5 **(E)** in liver tissue of mice in each group.

The protein results showed that, except for STAT5a, the expression trends of other proteins in the liver tissues of all groups were consistent: C > EM > CM (Figure 6). Compared to C group, the expression levels of PRLR and phosphorylated STAT5 (p-STAT5) were significantly reduced in both CM group and EM group (*P* < 0.01). However, only phosphorylated JAK2 (p-JAK2) in CM group showed a significant decrease compared to C group (*P* < 0.01). In contrast, the expression levels of PRLR, p-JAK2, and p-STAT5 in EM group were significantly higher than those in CM group (*P* < 0.01).

## 4 Discussion

The development and progression of NAFLD involve multiple factors, particularly its close association with metabolic disorders such as insulin resistance, hyperlipidemia, and obesity (Loomba et al., 2021; Wei et al., 2024; Peng et al., 2024). Among these, the “two-hit hypothesis” proposed by Day and James plays a significant role in the pathogenesis of NAFLD (Tan and Wang, 2017). This hypothesis suggests that the first hit originates from hepatic lipid metabolism disorders, leading to abnormal accumulation of triglycerides (TG) in hepatocytes and resulting in hepatic steatosis. The accumulation of TG in hepatocytes further increases the risk of various secondary hits, subsequently triggering non-alcoholic steatohepatitis (NASH) and fibrosis. Hepatic lipid deposition is a critical factor influencing the onset, progression, and outcomes of NAFLD (Mungamuri et al., 2021). Pan et al. (2016) demonstrated that feeding mice a high-fat diet for 8 weeks resulted in large and small lipid droplets scattered throughout the liver, visible fat vacuoles in the cytoplasm, and significant hepatocyte swelling with inflammatory features. Guo et al. (Yi-qiong et al., 2020) reported that a 10-week high-fat diet in rats induced abnormalities in liver enzymes, blood lipid levels, HOMA-IR, and hepatic reactive oxygen species content, accompanied by liver dysfunction, lipid metabolism disorders, insulin resistance, and oxidative stress. Ruan (2019) used a 16-week high-fat diet to induce abnormal lipid metabolism and oxidative stress indicators in rats, with pathological diagnosis by two professional pathologists confirming that the liver tissue HE staining met the criteria for clinical NAFLD patients. In this study, an 18-week high-fat diet successfully induced abnormal lipid metabolism in mice, as evidenced by significantly higher liver weight, liver index, TC, LDL-C, and LDL-C levels in the CM group compared to the C group (*P* < 0.01). HE staining revealed significant hepatic steatosis in the CM group, characterized by disordered hepatocyte arrangement, nuclei displaced to the cell periphery due to lipid droplet accumulation, scattered small fat vacuoles in the cytoplasm, blurred hepatic lobules, and disappearance of hepatic cords. Oil Red O staining showed minimal red staining in the C group, while the CM group exhibited extensive red-stained lipid droplets of varying sizes in the cytoplasm, indicating substantial lipid deposition in hepatocytes, consistent with previous findings. Based on the biochemical and pathological diagnostic criteria for NAFLD and related literature (Asgharpour et al., 2016), the 18-week high-fat diet successfully replicated the NAFLD model in mice.

Gao et al. (2020) reported that exercise intervention significantly reduced lipid droplet formation and decreased hepatic triglyceride accumulation in both *in vivo* and *in vitro* models of high-fat diet-induced NAFLD. In an animal study, Yang et al. (2022) found that exercise improved histological features of NAFLD, including hepatic steatosis, inflammation, and lobular ballooning, while preventing HFD-induced liver fat deposition and injury. A meta-analysis by Hashida et al. encompassing 12 studies revealed that both aerobic and resistance exercise effectively reduced hepatic steatosis in NAFLD patients, with no significant differences in frequency, duration, or exercise time between these two modalities (Hashida et al., 2017). Keating et al. (2015) conducted a clinical trial involving 48 overweight and obese patients, demonstrating that various aerobic exercise regimens significantly reduced liver fat content regardless of dose or intensity, although no substantial weight loss was observed. Furthermore, Katsagoni et al.‘s systematic review of 20 randomized clinical trials confirmed that exercise interventions positively impact intrahepatic triglyceride levels independent of weight reduction (Katsagoni et al., 2017). In the present study, we observed significant improvements in exercise-treated (EM group) mice compared to control (CM group) animals. The EM group exhibited significantly lower body weight, liver wet weight, liver index, and LDL-c levels (P < 0.01). Histopathological analysis revealed marked improvements in hepatic steatosis, with reduced lipid vacuoles around hepatocytes and nuclei in the EM group. Oil Red O staining demonstrated significantly fewer lipid droplets in EM group hepatocytes compared to the CM group. These findings collectively indicate that exercise intervention effectively reduces hepatic lipid deposition and ameliorates steatosis in NAFLD.

Although previous studies have demonstrated the beneficial effects of exercise on NAFLD through various mechanisms, the impact of aerobic exercise on circulating prolactin levels and its associated pathways in hepatic lipid metabolism in NAFLD mice remains unclear. We hypothesized that aerobic exercise may alleviate hepatic lipid accumulation by elevating circulating PRL levels in NAFLD mice. PRL is primarily secreted by the lactotroph cells of the anterior pituitary gland and is a member of the growth hormone/prolactin family. It primarily acts on the mammary glands as its target organ and is involved in various biological processes, including participation in substance metabolism, regulation of gonadal function, involvement in growth and development, response to stress, modulation of immune function, and regulation of electrolyte balance (Chasseloup et al., 2024). The concept of low circulating PRL levels as a clinical syndrome appeared for the first time in 2009 (Corona et al., 2009) in association with sexual dysfunction in which male patients with PRL serum levels b5 μg/L showed a higher risk of MS (Macotela et al., 2020; Corona et al., 2009). Zhang et al. (2023) also found that serum prolactin levels were significantly lower in obese patients with NAFLD, and a decreased serum prolactin level was associated with a significantly increased risk of NAFLD. We also observed that serum PRL levels in the CM group were significantly lower than those in C group but significantly higher than those in EM group, suggesting that exercise may moderately increase serum PRL levels in NAFLD mice. The metabolically beneficial effects of PRL occur directly on the target tissues via several molecular mechanisms that activate the PRLR canonical signaling pathway (Macotela et al., 2020), and JAK2/Stat5 pathway is considered the major downstream pathway for PRLR signaling (Chasseloup et al., 2024). JAK2 and STAT5 are critical for hepatic metabolic homeostasis, and the deficiency of either JAK2 or STAT5 disrupts the hepatic GH-JAK2-STAT5 signaling pathway, leading to significant lipid accumulation in hepatocytes, accompanied by increased peripheral lipid breakdown and enhanced hepatic lipogenesis, thereby promoting the development and progression of NAFLD (Kaltenecker et al., 2019). Our results revealed that PRLR and p-STAT5 were significantly reduced in CM group versus controls (P < 0.01). Aerobic exercise intervention (EM group) notably increased PRLR, p-JAK2, and p-STAT5 levels (P < 0.01), indicating that exercise may mitigate hepatic steatosis in NAFLD mice, likely through activation of the classical PRLR-mediated JAK2/STAT5 signaling pathway in liver.

Nevertheless, this study has several notable limitations. First, the exclusive use of male mice precludes the identification of potential sex-specific differences in both high-fat diet (HFD)-induced NAFLD pathogenesis and the physiological/cellular adaptations to treadmill training. Second, while we measured total STAT5a protein levels, we did not distinguish between the two STAT5 isoforms (STAT5a and STAT5b), which function as distinct transcription factors mediating diverse cytokine signaling pathways (Grimley et al., 1999). These limitations highlight the need for future mechanistic studies to comprehensively evaluate the multi-faceted metabolic pathways through which treadmill training ameliorates hepatic lipid deposition in NAFLD.

## 5 Conclusion

Exercise may moderately elevate serum prolactin (PRL) levels, thereby reducing intrahepatic lipid accumulation and ameliorating non-alcoholic fatty liver disease (NAFLD). The underlying mechanism may involve upregulation of the hepatic classical PRLR-mediated JAK2/STAT5 signaling pathway.

## Data Availability

The original contributions presented in the study are included in the article/supplementary material, further inquiries can be directed to the corresponding authors.
